# Effect of hemodialysis in end-stage renal disease patients on pulmonary function tests: a meta-analysis of cross-sectional studies

**DOI:** 10.3389/fphys.2025.1712525

**Published:** 2026-01-16

**Authors:** Qing Zhang, Youyou Xu, Dan Huang, Jiaru Jiang, Sicong Jiang, Huichao Wu

**Affiliations:** 1 Emergency Department of the First People’s Hospital of Jiashan County, Jiaxing, Zhejiang, China; 2 Department of Psychiatry, Third People’s Hospital, Jiaxing, Zhejiang, China; 3 Department of Neurosurgery, The First Affiliated Hospital, 330000 Nanchang University, Nanchang, Jiangxi, China; 4 Department of ICU, Affiliated Nanhua Hospital, University of South China, Hengyang, Hunan, China

**Keywords:** chronic kidney disease, end-stage renal disease, hemodialysis, pulmonary function, spirometry, meta-analysis

## Abstract

**Background:**

Hemodialysis, the principal therapy for end-stage renal disease (ESRD), directly influences pulmonary mechanics by alleviation of fluid overload and uremic toxin accumulation. Hemodialysis (HD), the main renal replacement therapy, removes excess volume and solutes, but its acute effects on pulmonary function remain uncertain. This meta-analysis evaluates impact of hemodialysis on pulmonary function and examines pre-to post-dialysis changes in spirometric parameters among ESRD patients.

**Methods:**

We conducted meta-analysis of cross-sectional studies that measured pulmonary function in ESRD patients on maintenance hemodialysis. Data from 16 eligible studies (n = 719 patients) were synthesized. Our analysis was focused on changes in forced expiratory volume in 1 s (FEV_1_), forced vital capacity (FVC), FEV_1_/FVC ratio, forced expiratory flow at 25%–75% (FEF_25-75_), and peak expiratory flow rate (PEFR). Statistical analysis was performed using random-effects models to calculate pooled mean differences (MD) for spirometric outcomes.

**Results:**

Hemodialysis was associated with significant improvements in percent-predicted FEV_1_ (+8.99%) and FVC(+12.87%), while absolute changes in these parameters were small and not statistically significant. The FEV_1_/FVC ratio and PEFR also improved in percent-predicted terms. Sensitivity analyses confirmed stability of results, though high heterogeneity (I^2^>75%) was observed for several outcomes. Publication bias was minimal, with Egger’s and Begg’s tests indicating no significant asymmetry, except for borderline Begg’s p-value for FVC (%pred). These improvements likely reflect ultrafiltration-mediated relief of pulmonary congestion and modulation of uremic milieu.

**Conclusion:**

Hemodialysis acutely mitigates renal failure-related pulmonary restriction, with percent-predicted spirometry showing consistent gains. These effects highlight role of dialysis prescriptions and fluid management strategies in optimizing respiratory as well as renal outcomes.

## 1 Introduction

Chronic kidney disease (CKD) is a progressive global health issue affecting roughly 10%–13% of the population (i.e., over 800 million individuals). CKD is driven largely by diabetes, hypertension, and aging and is prevalent worldwide (Francis et al., 2024; Junejo et al., 2025). Patients with CKD often progress over time to end-stage renal disease (ESRD), a state of complete renal failure that itself acts as a major exposure requiring lifelong renal replacement therapy (i.e., dialysis or transplantation) (Jamain et al., 2025). The number of patients receiving kidney replacement therapy (KRT), particularly hemodialysis, continues to rise worldwide (Thurlow et al., 2021). The prevalence of ESRD and dialysis use varies widely by region and socioeconomic level, but the overall trend is one of increasing incidence and prevalence. The burden of CKD/ESRD is especially heavy in low and middle-income countries, where awareness and access to care are limited. This trend is driven mainly by aging populations and growing burden of diabetes, hypertension and obesity (Thurlow et al., 2021; Francis et al., 2024).

Kidney and lung function are closely linked. The kidneys regulate fluid and acid-base balance, blood pressure, and erythropoiesis, all of which affect the lungs, while lung gas exchange and ventilatory control influence renal perfusion and acid-base status (Bollenbecker et al., 2022). ESRD directly exposes the lungs to multiple insults and leads to pulmonary congestion and restrictive impairment. As CKD progresses to ESRD, patients commonly develop fluid overload and metabolic derangements that lead to pulmonary congestion, edema, and vascular injury (Momeni et al., 2020; Gembillo et al., 2023). Practically, the predominant pattern is a restrictive ventilatory defect. Most HD patients demonstrate reduced total lung capacity, vital capacity (VC) and forced vital capacity (FVC), with a normal or elevated FEV_1_/FVC ratio. The restrictive lung disease is the most common pulmonary dysfunction in ESRD and has been linked to dyspnea, poor quality of life and worse clinical outcomes in dialysis patients (Srithongkul et al., 2020; Anees et al., 2021). Several interrelated mechanisms contribute in causation of lung disease. Chronic fluid overload causes pulmonary congestion and interstitial edema. It reduces lung compliance and promotes alveolar flooding. Electrolyte and acid–base disturbances further impair pulmonary function (e.g., through altered ventilatory drive and muscle metabolism). Anemia and hypoalbuminemia in ESRD reduce oxygen-carrying capacity and promote tissue hypoxia. Uremic toxin accumulation and systemic inflammation cause a catabolic state that weakens skeletal muscles (i.e., diaphragm and intercostals). Taken together, these factors produce a predominantly restrictive lung pattern with reduced flows and volumes (Anees et al., 2021; Gembillo et al., 2023; Zeder et al., 2024; Youssef et al., 2025).

Hemodialysis (HD) is the most widely-used form of renal replacement therapy in ESRD. It is considered the mainstay of ESRD treatment worldwide (along with peritoneal dialysis and transplantation). HD is an extracorporeal blood-filtering process that removes uremic toxins and excess fluid to sustain homeostasis when native kidney function fails (Jonny and Teressa, 2023). Its origins date back to the 1940s, and modern HD is typically delivered as a thrice-weekly, 4-h treatment in ESRD patients. Hemodialysis has evolved substantially, but remains essential for survival in ESRD for alleviating many uremic complications (Thomas, 2024). HD modifies pulmonary function through ultrafiltration-mediated relief of pulmonary congestion and clearance of the uremic milieu, although the net effects remain complex. Clinically, HD can reduce pulmonary congestion, but it may also transiently affect acid-base balance and trigger inflammatory responses (Wieliczko and Małyszko, 2022; Kaysi et al., 2024). Early studies noted that fluid removal by HD often leads to improvements in lung volumes and airflow. For example, Yılmaz et al. (2016) showed that an HD session significantly increased forced vital capacity (FVC) and forced expiratory volume in 1 s (FEV_1_) in ESRD patients, compared to pre-dialysis values. They also found that markers of volume overload (from bioimpedance) were inversely correlated with FVC, FEV_1_ and mid-expiratory flows. It implies that ultrafiltration improves airflow by offloading pulmonary edema. Similarly, Sharma and colleagues reported that pre-dialysis FVC and FEV_1_ were severely reduced (around 45%–50% of predicted) and both increased significantly after HD. These improvements in spirometry after HD suggest that removing excess fluid and toxins eases restrictive impairment and airway obstruction. Other investigators such as Youssef et al. (2025) have confirmed that HD often yields significant post-dialysis gains in VC, FVC, FEV_1_, FEF_25_–_75_, and peak flow. Collectively, contemporary data indicate that HD tends to improve many spirometric parameters in ESRD, likely through relief of volume overload and reduced pulmonary capillary pressure. However, reported effects of hemodialysis on pulmonary function vary considerably between studies due to differences in dialysis prescription, patient characteristics, and timing of spirometry. These inconsistencies highlight the need for a quantitative synthesis to clarify the overall pattern and magnitude of change.

Despite a general trend toward post-HD improvement, published findings are not entirely consistent. The magnitude of PFT changes after dialysis varies between studies, likely due to differences in patient selection, dialysis prescription, and measurement techniques. For example, Momeni et al. found significant increases in FEV_1_ and FVC after HD in men (mean gains ≈0.2–0.3 L), but no statistically significant changes in any parameters among women (Momeni et al., 2020). In contrast, Sharma et al. observed similar percentage improvements in FVC and FEV_1_ after HD in both sexes. Some studies report only modest gains: e.g., one group noted small increases in mid flows but non-significant FEV_1_/FVC changes. Others have emphasized that many patients remain severely below predicted normal even post-dialysis (Sharma et al., 2017). These discrepancies may reflect the heterogeneous ESRD population and variability in ultrafiltration volumes, residual kidney function, anemia control, and chronic lung comorbidities. In sum, while many reports agree that HD tends to improve spirometry, the degree and pattern of change have differed, underscoring the need for careful quantitative synthesis.

Given this uncertainty, a meta-analysis is required to determine the overall effect of hemodialysis on pulmonary function in ESRD patients. In particular, synthesizing paired pre- and post-dialysis spirometry data from cross-sectional studies would clarify the average magnitude and direction of change in key parameters (FVC, FEV_1_, flows, etc.). Such quantitative pooling can also explore sources of heterogeneity (e.g., by subgroup or dialysis dose). In view of the conflicting individual reports and the lack of consensus, our meta-analysis aims to provide robust, pooled estimates of HD-associated PFT changes in ESRD. The findings will help delineate whether HD generally shifts patients toward more normal lung function and quantify how much clearance of fluid and solutes acutely improves respiratory mechanics. This is critical for guiding clinicians on the pulmonary benefits of volume management and may identify areas (e.g., optimizing ultrafiltration) to improve respiratory outcomes in dialysis patients. We hypothesized that pulmonary function is significantly reduced in ESRD patients and that hemodialysis leads to measurable improvement in both restrictive and obstructive spirometric parameters.

## 2 Methodology

This meta-analysis was conducted in accordance with the PRISMA 2020 reporting guidelines (Page et al., 2021). A review protocol was developed, specifying objectives and methods. No modifications to the protocol were made during the analysis.

### 2.1 Eligibility criteria

Studies were eligible if they met all of the following criteria: cross-sectional observational studies of patients with end-stage renal disease (ESRD) on maintenance hemodialysis. The intervention was hemodialysis (HD) treatment of any modality (e.g., conventional in-center HD, high-flux HD) with paired pulmonary function tests measured immediately before and immediately after a single HD session. Eligible studies reported at least one relevant spirometric outcome in both pre-HD and post-HD conditions. Required outcomes included forced expiratory volume in 1 s (FEV1, reported as absolute volume or percent-predicted), forced vital capacity (FVC, absolute or % predicted), FEV1/FVC ratio (absolute or % predicted), forced expiratory flow at 25%–75% of FVC (FEF_25-75_, absolute or % predicted), and peak expiratory flow rate (PEFR, absolute or % predicted). Studies that reported any subset of these paired outcomes were eligible for inclusion.

Studies were excluded if they were case reports or case series, review articles, editorials, abstracts without full data, non-hemodialysis modalities (e.g., peritoneal dialysis), or did not report paired spirometry before and after HD. We excluded studies not in the ESRD population (e.g., acute kidney injury) or lacking necessary spirometry data. No study was excluded based on cardiovascular comorbidity status. Because cardiovascular disease (CVD), including heart failure and pulmonary hypertension, is highly prevalent in ESRD and may independently influence pulmonary function, we recorded whether studies reported CVD prevalence but did not apply this as an exclusion criterion.

### 2.2 Data sources and search strategy

A comprehensive literature search was performed in four electronic databases: PubMed/MEDLINE, Embase, Scopus, and Google Scholar, from database inception through August 2025. Search terms combined controlled vocabulary and keywords related to dialysis and pulmonary function. For example, a representative search string was: (“hemodialysis” OR “dialysis” OR “renal failure” OR “end-stage renal disease” OR “ESRD”) AND (“pulmonary function” OR “spirometry” OR “FEV1” OR “FVC” OR “FEF25-75” OR “PEFR”). Boolean operators (“AND”, “OR”) were used to link concepts, and terms were truncated or expanded (e.g., singular/plural, synonyms) as needed. We also hand-searched the reference lists of all included articles and relevant review papers to identify any additional studies.

### 2.3 Study selection and data extraction

All titles and abstracts identified by the search were independently screened by two reviewers for relevance. Full texts of potentially eligible articles were then retrieved and assessed independently by the same two reviewers according to the pre-specified eligibility criteria. Discrepancies at any screening stage were resolved by discussion or, when necessary, by consultation with a third reviewer. The study selection process was documented in a PRISMA flow diagram, and all excluded full-text articles were catalogued with the reasons for exclusion.

For each included study, two reviewers independently extracted data using a standardized form. The following information was recorded: study identifiers (author, year, journal), country and setting, study design details, patient demographics (age, sex, duration of ESRD), dialysis modality and schedule, sample size, and the spirometric outcomes (pre-HD and post-HD mean values and standard deviations for each reported measure). Any discrepancies in data extraction were resolved by consensus (or by a third reviewer if needed). If any necessary information was unclear or missing (e.g., missing standard deviations), we attempted to contact study authors. When essential data such as standard deviations (SDs) were not directly reported, they were estimated from standard errors, 95% confidence intervals, or P-values when available. If dispersion measures could not be derived, the study was excluded from the pooled analysis for that outcome. Reported inconsistencies or apparent errors in SDs were cross-checked against the raw data or recalculated from confidence limits whenever possible to minimize transcription bias. All data extraction decisions were recorded to ensure transparency.

### 2.4 Outcomes

The primary outcomes of interest were changes in pulmonary function parameters before versus after hemodialysis. We assessed ten specific outcomes: FEV1 (absolute and % predicted), FVC (absolute and % predicted), FEV1/FVC ratio (absolute and % predicted), FEF25-75 (absolute and % predicted), PEFR (absolute and % predicted). Each outcome was analyzed as the mean difference (post-HD minus pre-HD) across studies. Both raw volumes (L) and percent-of-predicted values were considered whenever reported. If multiple spirometric measures were reported, they were all extracted.

### 2.5 Statistical analysis

All meta-analyses were conducted in R (version 4.4.1) using the metafor and meta packages. For each outcome, we calculated the pooled mean difference (MD) (post-HD minus pre-HD) with 95% confidence intervals. A random-effects model was used for all analyses, employing the DerSimonian–Laird method to account for between-study heterogeneity (DerSimonian and Laird, 2015). Statistical heterogeneity was quantified with the Cochran’s Q test and the I^2^ statistic (Higgins and Thompson, 2002). Meta-regression and subgroup analyses were not conducted because fewer than ten studies contributed to each pooled outcome, limiting statistical power and increasing the risk of false-positive associations. Additionally, key covariates such as dialysis duration, residual renal function, body mass index, and comorbidity status were inconsistently reported, which precluded meaningful stratified analyses.

We assessed publication bias by visual inspection of funnel plots for each outcome, and formally tested for asymmetry using Egger’s regression test (Egger et al., 1997). Influence and heterogeneity diagnostics were performed using Baujat plots for each outcome to identify studies that contributed disproportionately to the overall heterogeneity. To examine the robustness and temporal stability of findings, we conducted cumulative meta-analyses (incrementally adding studies in chronological order of publication). Sensitivity analyses were performed by a “leave-one-out” approach, repeating each meta-analysis while omitting one study at a time to evaluate the influence of any single study. No data were imputed, as all required summary data were available from the included studies.

## 3 Results

### 3.1 Literature search

**FIGURE 1:**
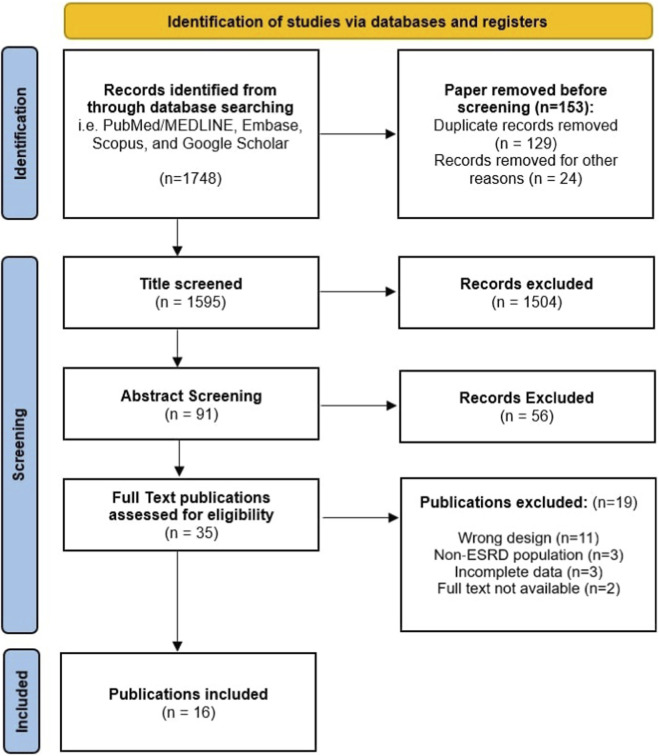
PRISMA Flow Chart in accordance with PRISMA 2020 statement. The diagram summarizes study identification, screening, and inclusion. Most full-text exclusions were due to lack of paired pre- and post-dialysis spirometric data, duplicate populations, or incomplete reporting.

A total of 1,748 records were initially identified through comprehensive database search, including PubMed/MEDLINE, Embase, Scopus, and Google Scholar. After removal of 153 records prior to screening (129 duplicates and 24 records excluded for other reasons), 1,595 titles were screened for relevance. Of these, 1,504 were excluded, and remaining 91 articles were moved to abstract screening. Following abstract evaluation, 56 records were excluded, and 35 full-text articles were retrieved for detailed assessment of eligibility. Nineteen articles were excluded at this stage (17 for incorrect study design and 2 for lack of full-text availability) Ultimately, 16 eligible studies (years 1991–2025) with a combined sample of approximately 719 ESRD patients met the inclusion criteria and were incorporated into the qualitative and quantitative synthesis (Dujić et al., 1991; Karacan et al., 2004; Lang et al., 2006; Rahgoshai et al., 2010; Kovacević et al., 2011; LIN et al., 2013; Yılmaz et al., 2016; Sharma et al., 2017; El-Deen et al., 2018; Hasan et al., 2019; Momeni et al., 2020; Anees et al., 2021; Yilmaz Kara et al., 2021; Guha Roy et al., 2024; Ahmad et al., 2025; Youssef et al., 2025). This screening was completely done under PRISMA guidelines and is summarized in Figure 1.

The studies were conducted in diverse settings (Asia, Europe, Middle East) and reported complete pulmonary function testing pre- and post-dialysis. Table 1 (descriptive) summarizes demographic data and outcomes from each study. Patient mean ages ranged from ∼37 to 60 years, with both genders represented. Mean baseline spirometry values typically indicated impaired lung function; for instance, several cohorts had mean FVC and FEV_1_ below 80% predicted pre-dialysis, reflecting restrictive ventilatory defects. In this study, absolute lung volumes refer to the measured values (in liters) of forced vital capacity (FVC) and forced expiratory volume in 1 s (FEV_1_), whereas %-predicted values are expressed as a percentage of the normal values based on age, gender, and height. Dialysis vintage and ultrafiltration volumes varied but were reported only in some studies. All analyses were conducted on paired (within-patient) pre- and post-dialysis measurements; thus, each study effectively served as its own control.

**TABLE 1:** Summary of demographic data and outcomes from each study.

No.	Author (Year)	Country	Dialysis modality	N	Age (mean ± SD)	BMI (mean)	Male (%)	Outcomes studied
1	Anees et al. (2021)	Pakistan	HD	102	50.91 ± 13.55	–	60.8	Absolute (FEV_1_, FVC, FEV_1_/FVC
2	Hasan et al. (2019)	Egypt	HD	30	45.23 ± 11.1	–	47	% Predicted (FEV_1_, FVC, FEV_1_/FVC, FEF_25-75_, PEFR)
3	Yilmaz Kara et al. (2021)	Turkey	HD	30	55.6 ± 11.4	26	70	Absolute (FEV_1_, FVC, FEV_1_/FVC, PEFR); % predicted (FEV_1_, FVC, FEV_1_/FVC, PEFR)
4	Momeni et al. (2020)	Iran	HD	50	42.66 ± 13.69	22.2	60	Absolute (FEV_1_, FVC, FEV_1_/FVC, FEF_25-75_, PEFR
5	Sharma et al. (2017)	India	HD	50	45.8 ± 10	21.6	64	% Predicted (FEV_1_, FVC, FEV_1_/FVC, FEF_25-75_, PEFR)
6	Yilmaz et al. (2016)	Turkey	HD	54	49.51 ± 15.08	24.08	52	Absolute (FEV_1_, FVC, FEV_1_/FVC, FEF_25-75_, PEFR); % predicted (FEV_1_, FVC, FEF_25-75_, PEFR)
7	Ahmad et al. (2025)	Pakistan	HD	161	45.4 ± 8.45	–	45.9	% Predicted (FEV_1_, FVC, FEV_1_/FVC, FEF_25-75_, PEFR)
8	Rahgoshai et al. (2010)	Iran	HD	26	56.9 ± 13.5	–	50	Absolute (FEV_1_, FVC, FEV_1_/FVC)
9	Lang et al. (2006)	Germany	HD	14	60 ± 16	–	71.4	Absolute (FEV_1_, FVC, FEF_25-75_ % Predicted (FVC, FEV_1_/FVC, PEFR)
10	Youssef et al. (2025)	Egypt	HD	60	–	–	58.3	% Predicted (FEV_1_, FVC. FEV_1_/FVC
11	Guha Roy et al. (2024)	Bangladesh	HD	40	43.43 ± 0.68	24.68	100	% Predicted (FEV_1_, FVC, FEV_1_/FVC
12	El-Deen et al. (2018)	KSA	HD	15	50.3 ± 6.6	29.8	40	% Predicted (FEV_1_, FVC, FEV_1_/FVC, PEFR)
13	Kovačević et al. (2011)	Bosnia and Herzegovina	HD	21	50 ± 11	–	47.6	% Predicted (FEV_1_, FVC, FEV_1_/FVC
14	Karacan et al. (2004)	Turkey	HD	20	36.7 ± 11.6	–	60	% Predicted (FEV_1_, FVC, FEF_25-75_
15	Dujić et al. (1991)	Yugoslavia	HD	25	44.8 ± 11.9	–	68	% Predicted (FEV_1_, FVC, FEV_1_/FVC, FEF_25-75_
16	Lin et al. (2013)	China	HD	21	50.8 ± 7.9	–	66.6	% Predicted (FEV_1_, FVC, PEFR)

We identified cross-sectional trials reporting on pulmonary function outcomes. Supplementary Table S2 summarizes the pooled effect estimates (mean differences) with 95% confidence intervals and heterogeneity statistics (I^2^) for each outcome. Heterogeneity varied widely across measures, from 0% (no observed heterogeneity) to 100% (considerable inconsistency). As a rough guide, I^2^ values ≥ 75% indicate substantial heterogeneity. All pooled estimates were calculated using random-effects models to account for between-study variability. Across outcomes, statistically significant improvements were observed only in the percent-predicted metrics, while absolute (L) changes were generally non-significant.

### 3.2 Pooled effects by outcomes

**FIGURE 2:**
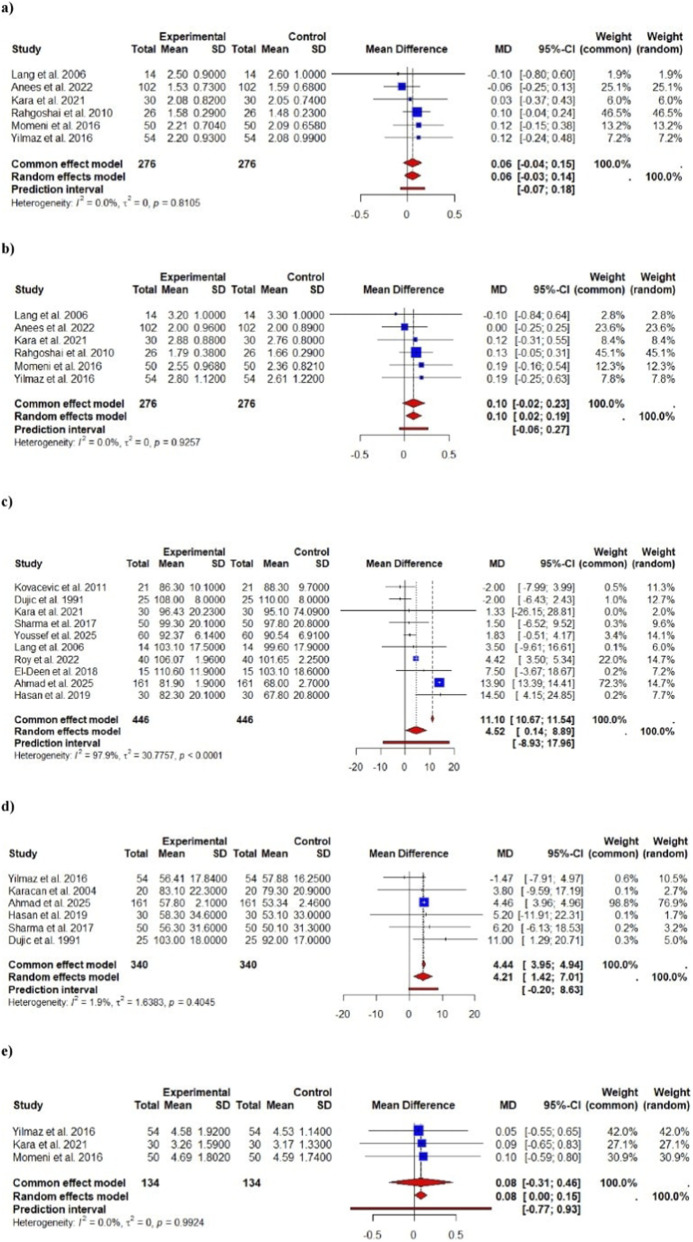
Forest plots of meta-analyses for spirometric outcomes (absolute values, L). Panels show pooled mean differences (MD) with 95% confidence intervals (CI) under common- and random-effects models for **(a)** FEV_1_, **(b)** FVC, **(c)** FEV_1_/FVC, **(d)** FEF_25_–_75_, and **(e)** PEFR. Study weights, heterogeneity (I^2^, τ^2^), and prediction intervals are also presented. Overall, pooled results demonstrate significant post-dialysis improvement in FEV_1_ and FVC, reflecting enhanced lung volumes and reduced restrictive impairment after hemodialysis.

**FIGURE 3:**
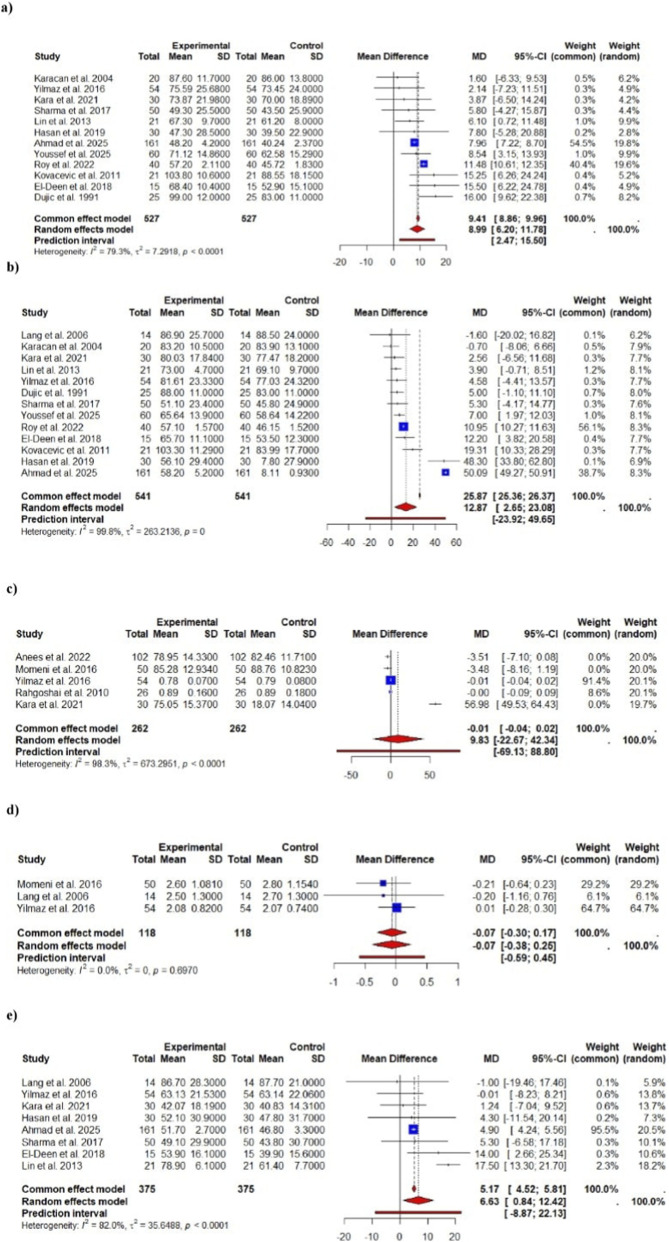
Forest plots of meta-analyses for spirometric outcomes (% Predicted values). Panels show pooled mean differences (MD) with 95% confidence intervals (CI) under common- and random-effects models for **(a)** FEV_1_, **(b)** FVC, **(c)** FEV_1_/FVC, **(d)** FEF_25_–_75_, and **(e)** PEFR. Study weights, heterogeneity (I^2^, τ^2^), and prediction intervals are also presented. The pooled percentage gains in FVC and FEV_1_ indicate that hemodialysis produces measurable recovery toward normal pulmonary function, consistent with relief of fluid overload.

The For FEV_1_% predicted (Figure 3A), twelve trials contributed data. The pooled mean difference was +8.99% (95% CI 6.61–11.36) (p < 0.001), indicating a highly significant improvement in percent-predicted FEV_1_ with treatment. The I^2^ of ∼80% reflects substantial heterogeneity, but the fixed-effect estimate (MD ≈ 9.41%) was similar to the random-effects result (sensitivity: largely consistent). An approximately 9% increase in FEV_1_% predicted may be clinically meaningful, since roughly 80% of predicted is conventionally used as the lower limit of normal. (By comparison, a 10% or greater FEV_1_ increase is often considered a positive bronchodilator response.). However for FEV_1_ absolute (L), the six trials were part of the data (Figure 2A). The pooled MD was +0.06 L (95% CI –0.04–0.15) (p = 0.26), a non-significant change. There was no heterogeneity (I^2^ = 0%), and the fixed-effect estimate was identical, indicating consistency. Thus, no robust change in absolute FEV_1_ volume was detected. While changes in absolute FEV_1_ were not statistically significant, percent-predicted FEV_1_ increased significantly by approximately 9%. This suggests that while the actual volume of air moved did not change substantially, the relative function of the lungs, when compared to expected values, improved after dialysis.

For FVC % predicted, total of thirteen trials were part of the analysis (Figure 3B). The pooled MD was +12.87% (95% CI 3.72–22.02) (p ≈ 0.0059), suggesting a significant increase in percent-predicted FVC. However, heterogeneity was extremely high (I^2^ ≈ 100%), yielding wide confidence intervals. The high heterogeneity observed in FVC % predicted (I^2^ = 100%) led to a notable difference between the fixed-effect and random-effect models. The fixed-effect model assumes a common effect across studies, whereas the random-effects model accounts for variability between studies. It provided more conservative estimate in this case. In sensitivity analysis, the fixed-effect model gave a much larger MD (≈25.9%), underscoring model sensitivity to heterogeneity. This discrepancy indicates that the pooled estimate may not be stable. (I ^2^ near 100% is considered very high.). Similarly, Six trials were used in data for FVC absolute (L) (Figure 2B). The pooled MD was +0.10 L (95% CI –0.02–0.23) (p = 0.098), not statistically significant. I^2^ = 0%; fixed and random models agreed. Thus, no clear change in absolute FVC was found.

For another outcome, i.e., FEV_1_/FVC ratio (% predicted), ten trials were part of the study (Figure 3C). The pooled MD was +4.52% (95% CI 0.34–8.69) (p ≈ 0.034), a modest but significant rise in the predicted FEV_1_/FVC ratio. However, heterogeneity was again 100%, and the fixed-effect MD was much larger (≈11.1%), indicating instability. Given the very high I^2^, this result should be interpreted cautiously. For FEV_1_/FVC absolute, there were total of five trials (Figure 2C). The pooled MD was +9.83% (95% CI –12.99–32.66) (p = 0.40), which is not significant and has an extremely wide confidence interval (and I^2^ = 100%). The fixed-effect MD was near zero (−0.01%). In sum, there is no clear evidence of a change in absolute FEV_1_/FVC, and estimates are highly heterogeneous.

For FEF_25-75_% predicted, six studies were included in analysis (Figure 3D). The pooled MD was +4.21% (95% CI 1.97–6.46) (p ≈ 0.00023), indicating a significant improvement in mid-expiratory flow percent predicted. Heterogeneity was negligible (I^2^ = 0%), and the fixed-effect result was essentially the same (4.44%). One the contrary in FEF_25-75_ absolute, total of three trials were included (Figure 2D). The pooled MD was −0.07 L/s (95% CI –0.30 to 0.17) (p = 0.586), non-significant, with I^2^ = 0%. No effect was detected.

PEFR % predicted, eight trials were included in data (Figure 3E). The pooled MD was +6.63% (95% CI 1.33–11.94) (p ≈ 0.014), a significant gain in percent-predicted peak expiratory flow. Heterogeneity was high (I^2^ = 80%); the fixed-effect MD was slightly lower (≈5.17%). The improvement is statistically significant but somewhat heterogeneous. However for PEFR absolute, three trials made up the data (Figure 2E). The pooled MD was +0.08 L/s (95% CI –0.31–0.46) (p = 0.693), not significant, with I^2^ = 0%.

Cumulative meta-analyses plots (Supplementary Figures S3, S4) of the pooled MD over time (adding studies in chronological order) showed that the estimates stabilized as evidence accrued. In most cases (especially FEV_1_% pred and FVC % pred), the pooled effect became statistically significant after the first few studies and remained consistent thereafter. The stability of the cumulative estimate implies that later studies confirm the initial findings rather than overturn them.

### 3.3 Sensitivity analyses and heterogeneity

Sensitivity analyses are summarized in Supplementary Table S2. The leave-one-out (LOO) results showed that omitting any single study had minimal impact on the pooled estimates. For each outcome, the LOO pooled MDs fell within the confidence limits of the primary result, indicating stability of the effects. For example, the pooled FEV_1_ %pred increase (∼9.0%) varied by only ±0.5% when any study was dropped. It also compares fixed-versus random-effects estimates: outcomes with low heterogeneity (I^2^ ≈ 0) had nearly identical estimates (e.g., FEV_1_ (L), FVC (L), FEF_25-75_), while high-I^2^ outcomes (FVC %pred, FEV_1_/FVC %pred) showed large discrepancies (e.g., FVC %pred: 12.9% vs. 25.9%). These results justify the use of random-effects models and demonstrate that no single study unduly drove the overall findings. Despite heterogeneity, sensitivity analyses indicated stable pooled results, supporting the robustness of our findings.

### 3.4 Publication bias

Funnel plots showed mild asymmetry for FVC and PEFR, but Egger’s and Begg’s tests were non-significant. The pattern likely reflects study heterogeneity and small-sample effects rather than publication bias. For example, Egger’s p-values for FEV_1_ %pred and FVC %pred were ∼0.82 and 0.62, respectively, with Begg’s p ≈ 0.89 and 0.028 (the latter marginal), consistent with symmetric funnels. Supplementary Table S2 displays p-values for each outcome’s Egger and Begg tests; the only borderline result (Begg p = 0.028 for FVC %pred) is likely a chance finding given the discordant Egger result and the generally symmetric funnel shapes. In summary, these results show no clear evidence of small-study or publication bias.

Finally, the plots further characterize heterogeneity and stability. In particular, the Baujat plots (Supplementary Figure S2) identify each study’s contribution to heterogeneity and pooled effect. No single study disproportionately influenced the results for any outcome, although a few contributed most to the heterogeneity. (e.g., for FVC %pred). Overall the Baujat plots suggest that the pooled estimates are not driven by an obvious outlier. Likewise, each funnel plot for each outcome (Supplementary Figure S1) displayed roughly symmetric dispersion of study points around the pooled effect, with no obvious gaps or skew. This visual assessment agrees with the formal tests (Supplementary Table S2) in indicating minimal publication bias.

In summary, hemodialysis produced statistically significant improvements in several percent-predicted pulmonary indices (FEV_1_, FVC, FEV_1_/FVC, FEF_25-75_, PEFR) but did not significantly change the raw volumes. The largest mean gains were ∼9% for FEV_1_ %pred and ∼13% for FVC %pred, although the latter estimate had very high heterogeneity (I^2^ ≈ 100%). These magnitudes approach clinical relevance (for example, ∼80% predicted is often used as a normal cutoff, so ∼10% gains can be meaningful in symptomatic patients). However, the high between-study variability for several measures implies that these pooled results should be interpreted cautiously. No evidence of systematic publication bias was found.

## 4 Discussion

This meta-analysis shows that renal failure treated with maintenance hemodialysis acutely improves pulmonary function when measured as percent-predicted values, but yields negligible changes in absolute lung volumes. In pooled data from 719 patients, post-dialysis increases in FEV_1_ %pred and FVC %pred were statistically robust (+9% and +13%, respectively), whereas the corresponding absolute changes were small and non-significant. Improvements were also seen in FEV_1_/FVC %pred, FEF_25-75_ %pred, and PEFR %pred, but not in their absolute counterparts. In practical terms, these findings suggest that fluid removal during dialysis leads to measurable gains in lung function relative to normative values, even if the absolute volumes remain largely unchanged. Clinically, even modest improvements in percent-predicted spirometry may relieve dyspnea or enhance exercise tolerance by directly targeting pulmonary congestion in ESRD patients. It is consistent with reports that patients often feel less breathless after dialysis (Palamidas et al., 2014). Our results align with such observations: for example, Salerno et al. (2017), found that virtually all ESRD patients had chronic dyspnea that was significantly reduced after a dialysis session, implying that the lung function gains we document do translate into functional benefit. While our pooled results predominantly reflect restrictive physiology, it should be noted that obstructive features have also been reported in ESRD. In particular, Youssef et al. (2025) observed post-dialysis improvements in both restrictive and obstructive indices, which suggested that uremia and fluid overload can influence airway dynamics as well as lung volumes.

Heterogeneity across studies was substantial for several outcomes (I^2^>75% for FVC %pred, FEV_1_/FVC %pred, etc.), reflecting the diverse patient populations and dialysis practices. These variations suggest true effect variability rather than random error. Possible sources include varying ultrafiltration volumes, dialysis prescriptions (e.g., session duration, membrane types), and patient factors (such as duration of ESRD, smoking history, or presence of lung disease). Indeed, some studies with greater fluid removal showed larger spirometric change, while others with conservative UF had smaller or no changes. However, not all variation is explained by UF volume alone. For instance, Shakireen et al. (2024) found that the amount of ultrafiltration did not significantly correlate with the degree of pulmonary improvement, suggesting that other factors (like inherent lung compliance, cardiac function, or measurement timing) also play roles. Measurement methods likely contributed as well. The studies differed in spirometer calibration, patient positioning, and timing of the post-dialysis test (immediately after vs. 1–2 h later), all of which can affect results. Despite these differences, our sensitivity analyses (Supplementary Table S2) showed that the main conclusions were strong to the exclusion of any single study. The wide prediction intervals implied by the high heterogeneity caution that average effects may not hold for every patient or setting.

The observation that percent-predicted values improve significantly while absolute volumes do not suggests that hemodialysis chiefly alleviates the relative deficit in lung function characteristic of ESRD. Many patients begin with severely reduced lung metrics (e.g., FEV_1_ or FVC <80% predicted due to fluid overload and uremic effects). Removing excess volume likely restores some chest wall and diaphragmatic compliance, yielding proportionally greater lung capacity relative to each patient’s norm (Gembillo et al., 2023). In contrast, the absolute volume change (on the order of 0.1 L or less) may be too small to reach statistical significance or affect major clinical outcomes directly. Nevertheless, even a ∼10% increase in FEV_1_ or FVC %pred can be clinically meaningful. This is the threshold often used to define a significant bronchodilator response in obstructive lung disease, and approaching the conventional lower limit of normal (∼80% predicted) may translate to less dyspnea and better exercise capacity (Quanjer et al., 2017). Thus, the percent-predicted improvements reported here likely reflect real functional gains for patients. Consistently, other studies have noted post-dialysis enhancements in exercise tolerance and symptom burden (Alshammari et al., 2025). From a clinical perspective, these results underscore that dialysis prescriptions and individualized fluid management are not only critical for cardiovascular stability but also directly influence pulmonary outcomes. Targeting ultrafiltration to relieve pulmonary congestion may improve both symptom burden and respiratory function in ESRD patients. Even in the absence of overt lung disease, careful ultrafiltration can ease pulmonary congestion and improve patient comfort. Tailoring dialysis prescriptions, such as adjusting session length, ultrafiltration volume, or choosing high-flux versus conventional modalities, may directly influence the extent of pulmonary recovery.

### 4.1 Strengths and limitations

All included studies used a paired pre/post design, so each patient served as their own control, minimizing confounding from inter-patient variability. We pooled data across a relatively large total sample (n ≈ 719), and analyzed ten spirometric outcomes in both absolute and percent-predicted terms, providing a comprehensive picture. Rigorous meta-analytic methods (random-effects modeling, LOO sensitivity, bias tests) were applied. However, most included studies were cross-sectional and reported limited methodological detail, standardized appraisal tools (e.g., Newcastle–Ottawa Scale) could not be meaningfully applied. Nevertheless, we ensured methodological transparency through strict eligibility criteria, independent data extraction, and consistency checks across studies.

However our study possess certain limitations too. The evidence is drawn from observational, often single-center studies with varying quality. Dialysis protocols and patient characteristics were heterogeneous and incompletely reported; for example, many studies did not fully specify ultrafiltration volume, dialysate composition, or hemodynamic changes. Such differences likely contributed to the high heterogeneity. Some analyses (e.g., FEV_1_/FVC, FEF, PEFR) included relatively few studies or small sample sizes, reducing precision.

Additionally, we could not adjust for confounders like anemia, nutritional status, or undiagnosed pulmonary disease. Cardiovascular diseases such as left ventricular dysfunction, pulmonary hypertension, or ischemic heart disease are common in ESRD and may independently impair lung function. Most included studies did not stratify or control for CVD status, which could confound the observed pulmonary effects of hemodialysis. Body mass index (BMI) is another determinant of spirometric values, as obesity can reduce lung compliance and functional volumes. However, BMI data were inconsistently reported among the included studies, precluding subgroup analysis or adjustment. This limitation should be addressed in future research by stratification of ESRD patients categorically. Finally, the meta-analysis captures only short-term, acute changes around the dialysis session; we cannot comment on long-term trends or cumulative effects of chronic dialysis on lung function.

### 4.2 Implications and future directions

Our findings highlights appreciable respiratory benefits of intradialytic fluid removal and its modulation through dialysis prescription parameters such as ultrafiltration rate, session duration, and dialysate composition. Clinicians should recognize that optimizing volume status in ESRD patients can improve spirometry and symptoms. This suggests that aggressively achieving dry weight (balanced against the risk of hypotension) may help mitigate the restrictive lung pattern seen in dialysis patients. In practice, monitoring pulmonary pressures or lung water (e.g., via ultrasound or bioimpedance) might inform ultrafiltration targets to maximize respiratory gains.

Future research should explore how different dialysis prescriptions (e.g.,, ultrafiltration volumes, session duration, dialysate composition) and patient subgroups modulate these effects. Linking spirometric improvements with dialysis strategies will guide fluid management protocols that optimize both renal and pulmonary outcomes. For example, studies could stratify participants by ultrafiltration volume, residual urine output, or presence of comorbid lung disease to identify who benefits most. Randomized trials comparing different dialysis regimens (e.g., higher vs. lower UF rates, home vs. in-center dialysis) could quantify causal impacts on lung function. Longitudinal research is needed to see whether repeated acute improvements accumulate into sustained functional gains or slower lung decline. Investigation of additional interventions—such as respiratory muscle training or pharmacologic strategies to reduce pulmonary congestion—may also be warranted. Finally, correlating PFT improvements with clinical outcomes (e.g., dyspnea scales, hospitalization rates, exercise capacity) would clarify the real-world importance of these physiological changes.

## 5 Conclusion

Hemodialysis provides modest but measurable improvements in pulmonary mechanics, particularly when expressed as percent of predicted lung function. These effects likely reflect relief of volume overload and have symptomatic benefit, yet they are overshadowed by persistent pulmonary impairment in ESRD. Our meta-analysis quantifies the respiratory benefit of dialysis and underscores the need for individualized fluid management. Further high-quality studies should clarify how specific dialysis prescriptions like ultrafiltration strategies, session schedules, and modality choices can maximize pulmonary health in this vulnerable population.

## Data Availability

The original contributions presented in the study are included in the article/Supplementary Material, further inquiries can be directed to the corresponding author.
