# Supplementary material for: Effect of hemodialysis in end-stage renal disease patients on pulmonary function tests: a meta-analysis of cross-sectional studies
Source: Front Physiol. 2026 Jan 16;16:1712525. doi: 10.3389/fphys.2025.1712525 (PMC12855113; doi:10.3389/fphys.2025.1712525)
Supplement: Supplementary file 1 [file Supplementaryfile1.docx]

Supplementary Material

# Supplementary Figures

## Supplementary Figures

**
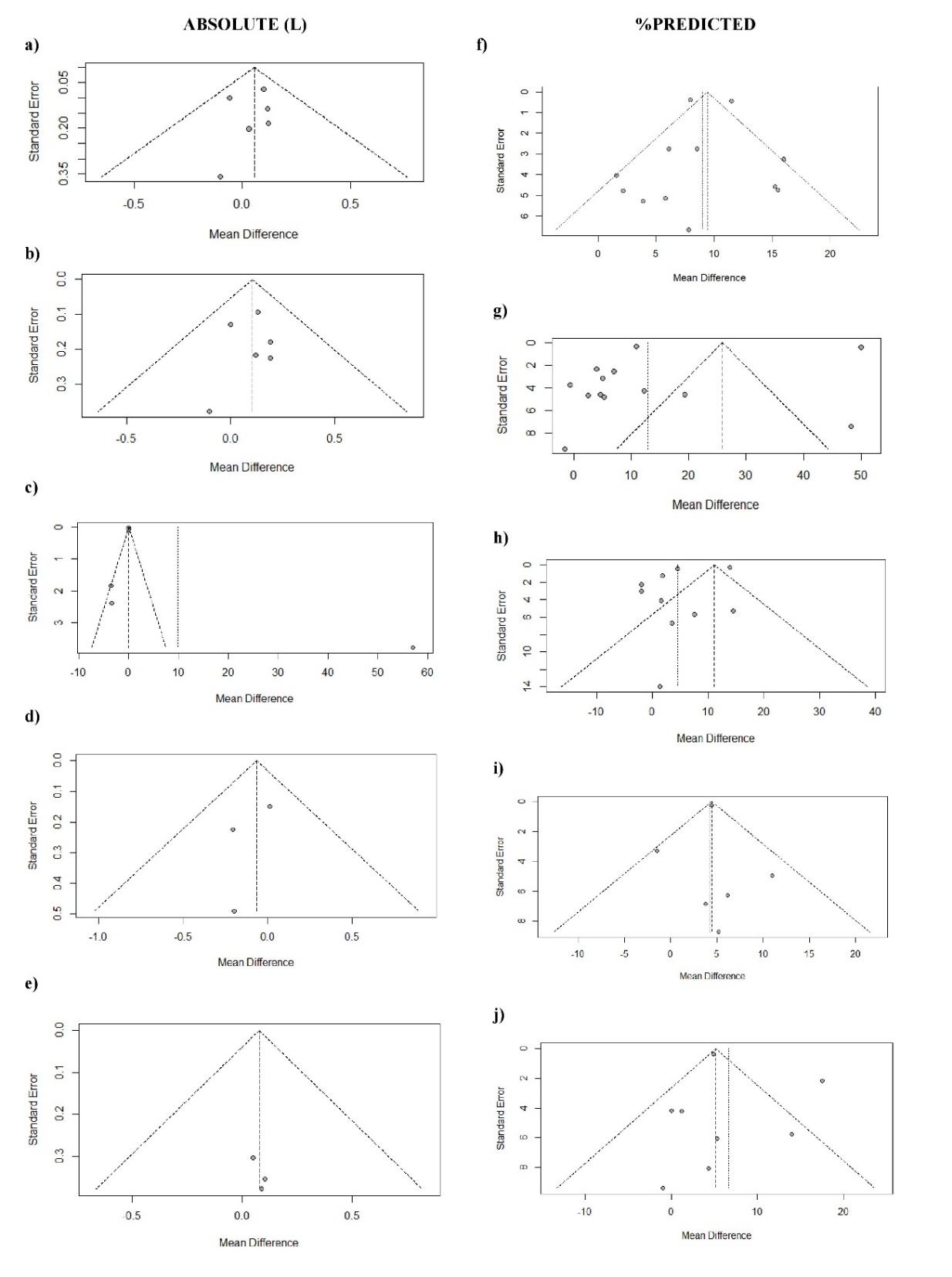
**

**Fig S1: Funnel plots assessing publication bias in meta-analyses of spirometric outcomes. Absolute values (L) are shown for (a) FEV₁, (b) FVC, (c) FEV₁/FVC, (d) FEF₂₅–₇₅, and (e) PEFR; % predicted values are shown for (f) FEV₁, (g) FVC, (h) FEV₁/FVC, (i) FEF₂₅–₇₅, and (j) PEFR.**

**
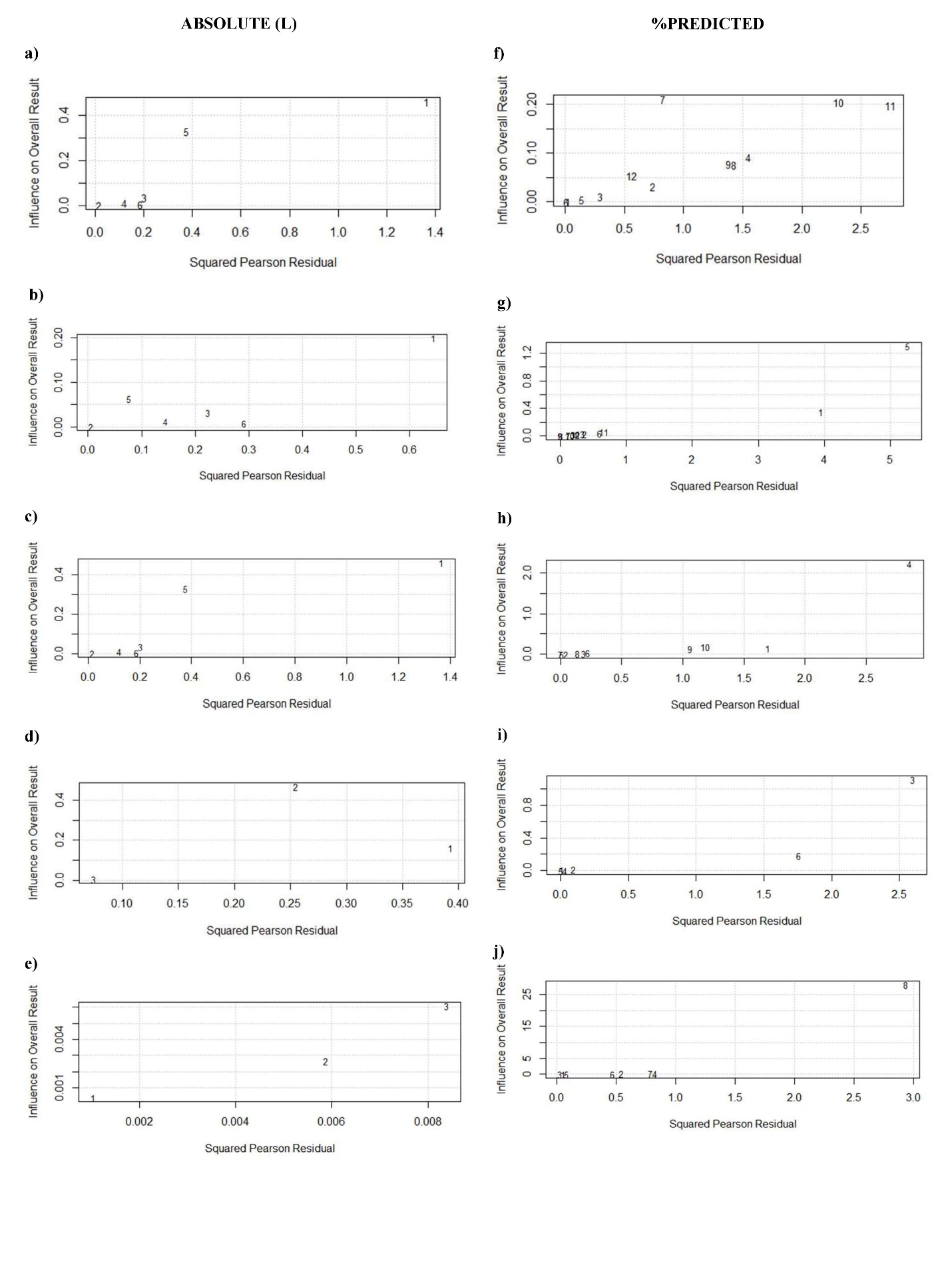
**

**Fig S2. Baujat plots showing the influence of individual studies on heterogeneity and overall effect size in meta-analyses of spirometric outcomes. Absolute values (L) are shown for (a) FEV₁, (b) FVC, (c) FEV₁/FVC, (d) FEF₂₅–₇₅, and (e) PEFR; % predicted values are shown for (f) FEV₁, (g) FVC, (h) FEV₁/FVC, (i) FEF₂₅–₇₅, and (j) PEFR.**

**
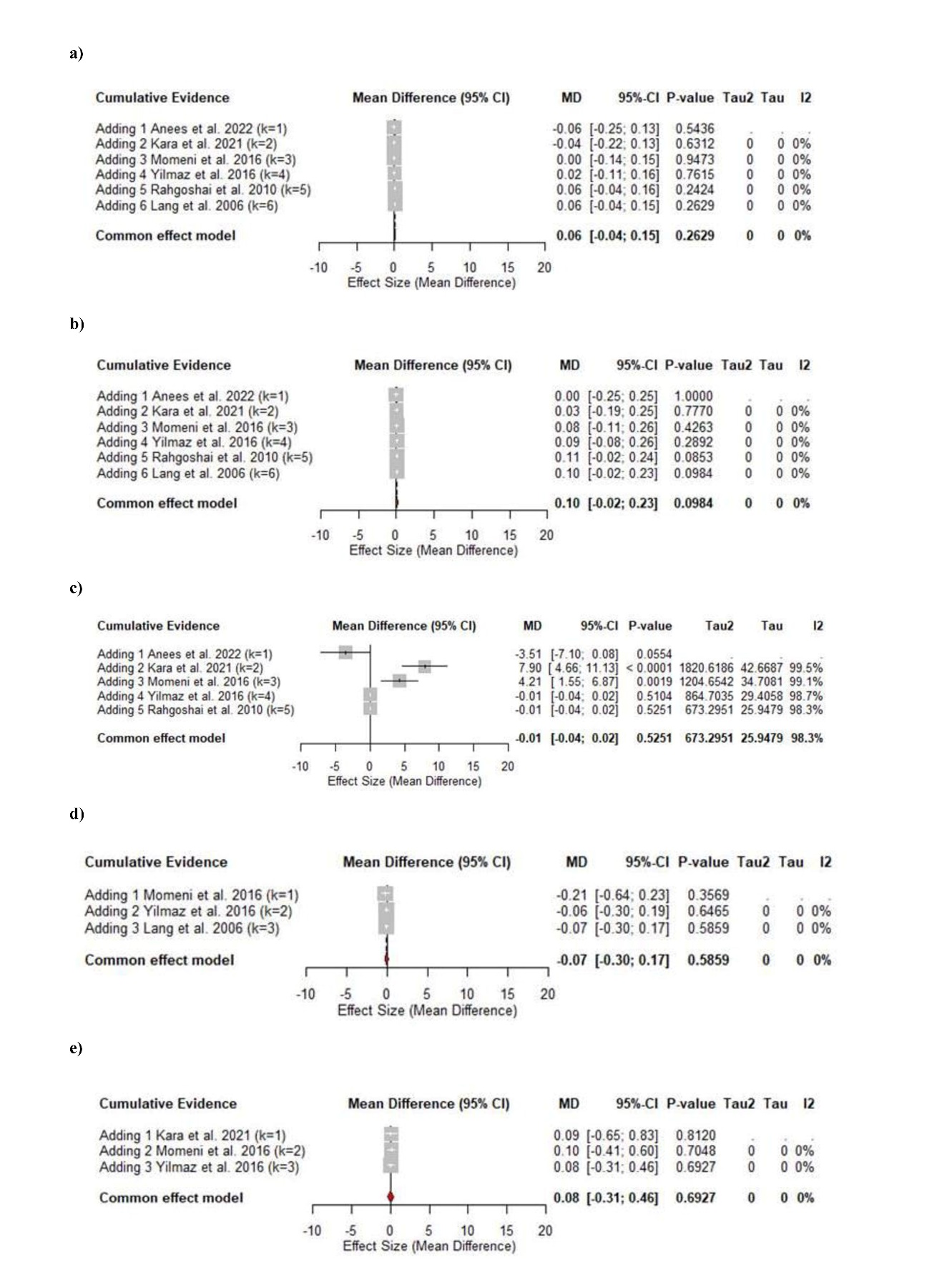
**

**Fig S3. Cumulative meta-analyses of spirometric outcomes (absolute values, L). Plots show sequential pooling of studies for (a) FEV₁, (b) FVC, (c) FEV₁/FVC, (d) FEF₂₅–₇₅, and (e) PEFR, under a common-effects model, with mean differences (MD), 95% confidence intervals (CI), and heterogeneity estimates (I², τ²).**

**
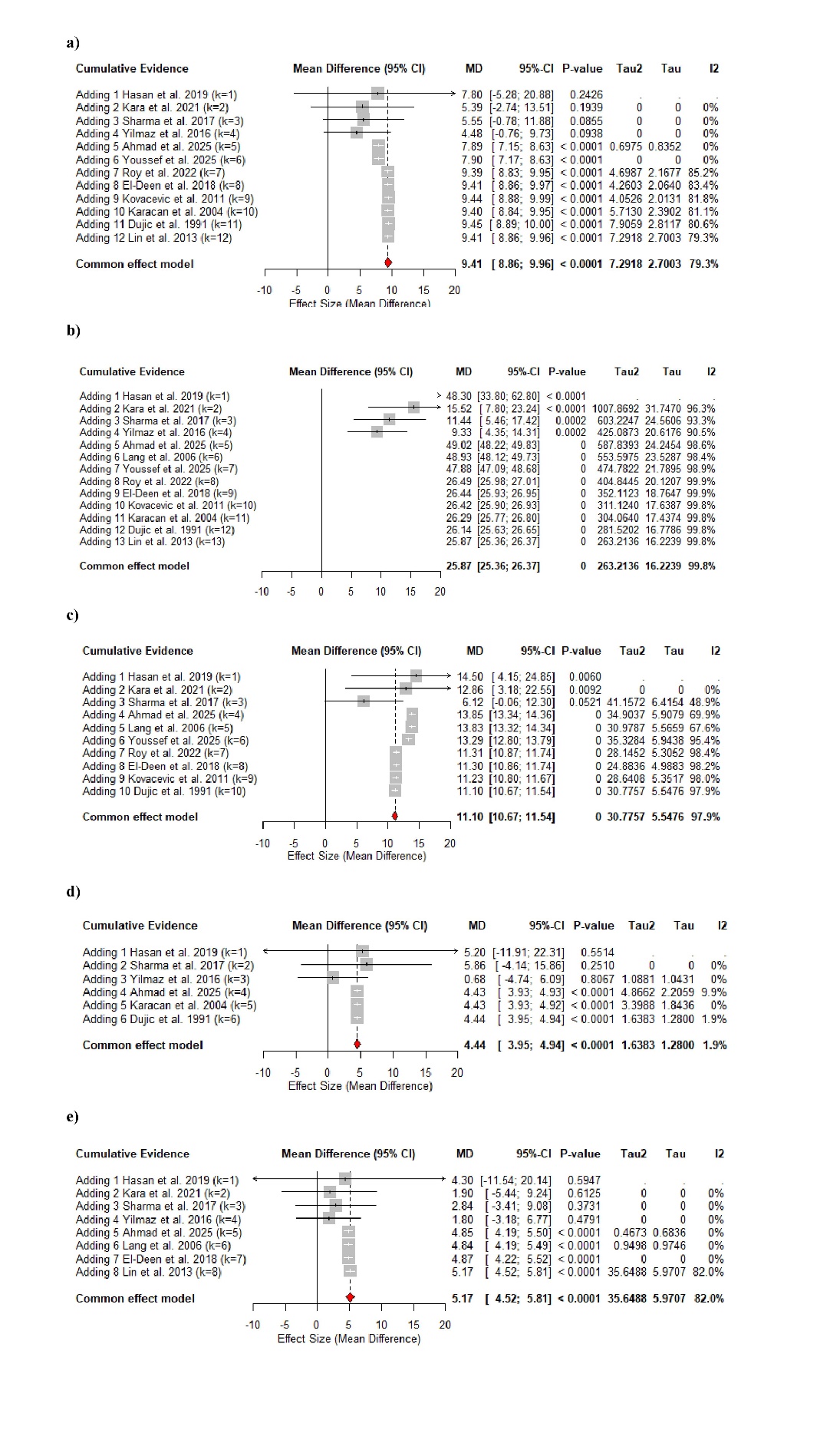
**

**Fig S4. Cumulative meta-analyses of spirometric outcomes (%Predicted values). Plots show sequential pooling of studies for (a) FEV₁, (b) FVC, (c) FEV₁/FVC, (d) FEF₂₅–₇₅, and (e) PEFR, under a common-effects model, with mean differences (MD), 95% confidence intervals (CI), and heterogeneity estimates (I², τ²).**
