# Supplementary material for: Effect of hemodialysis in end-stage renal disease patients on pulmonary function tests: a meta-analysis of cross-sectional studies
Source: Front Physiol. 2026 Jan 16;16:1712525. doi: 10.3389/fphys.2025.1712525 (PMC12855113; doi:10.3389/fphys.2025.1712525)
Supplement: Supplementary file 2 [file Table1.docx]

Supplementary Material

# Supplementary Table

**Table S1: Sensitivity analyses of meta-analytic results for spirometric outcomes, showing pooled mean differences (MD), heterogeneity (I²), leave-one-out (LOO) ranges, and publication bias tests (Egger’s, Begg’s).**

| **Outcome** | **Meta-analytic results for spirometric outcomes** | | | | | **Sensitivity analyses** | | | | **Funnel plot asymmetry (publication bias)** | |
| --- | --- | --- | --- | --- | --- | --- | --- | --- | --- | --- | --- |
|  | **Studies (k)** | **Mean diff (random) [95% CI]** | **p-value** | **I² (%)** | **τ²** | **Random-effects MD (95% CI)** | **Fixed-effects MD (95% CI)** | **I² (%)** | **LOO MD range** | **Egger’s p** | **Begg’s p** |
| FEV₁ (predicted, %) | 12 | 8.99 (6.61, 11.36) | 1.23x10^-13^ | 80 | 7.292 | +8.99 (6.61, 11.36)    % | +9.41 (7.20, 11.62)    % | 80 | 8.5% – 9.5% | 0.82 | 0.89 |
| FEV₁ (absolute, L) | 6 | 0.06 (-0.04, 0.15) | 0.263 | 0 | 0 | +0.06 (–0.04, 0.15) L | +0.06 (–0.04, 0.15) L | 0 | 0.05 – 0.07 L | 0.76 | 0.68 |
| FVC (predicted, %) | 13 | 12.87 (3.72, 22.02) | 0.00585 | 100 | 263.214 | +12.87 (3.72, 22.02)    % | +25.9 (20.0, 31.8)    % | 100 | 8% – 18% | 0.62 | 0.028* |
| FVC (absolute, L) | 6 | 0.10 (-0.02, 0.23) | 0.0984 | 0 | 0 | +0.10 (–0.02, 0.23) L | +0.10 (–0.02, 0.23) L | 0 | 0.08 – 0.12 L | 0.59 | 0.70 |
| FEV₁/FVC (predicted, %) | 10 | 4.52 (0.34, 8.69) | 0.0341 | 100 | 30.776 | +4.52 (0.34, 8.69)    % | +11.1 (7.5, 14.7)    % | 100 | 2% – 9% | 0.48 | 0.33 |
| FEV₁/FVC (absolute) | 5 | 9.83 (-12.99, 32.66) | 0.398 | 100 | 673.295 | +9.83 (–12.99, 32.66) | –0.01 (–2.50, 2.48) | 100 | –10% – 30% | 0.80 | 0.75 |
| FEF₂₅₋₇₅ (predicted, %) | 6 | 4.21 (1.97, 6.46) | 0.000232 | 0 | 1.638 | +4.21 (1.97, 6.46)    % | +4.44 (2.20, 6.68)    % | 0 | 3% – 5% | 0.53 | 0.61 |
| FEF₂₅₋₇₅ (absolute) | 3 | -0.07 (-0.30, 0.17) | 0.586 | 0 | 0 | –0.07 (–0.30, 0.17) L | –0.07 (–0.30, 0.17) L | 0 | –0.08 – 0.07 L | 0.92 | 0.81 |
| PEFR (predicted, %) | 8 | 6.63 (1.33, 11.94) | 0.0143 | 80 | 35.649 | +6.63 (1.33, 11.94)    % | +5.17 (0.75, 9.59)    % | 80 | 5% – 8% | 0.45 | 0.64 |
| PEFR (absolute) | 3 | 0.08 (-0.31, 0.46) | 0.693 | 0 | 0 | +0.08 (–0.31, 0.46) L | +0.08 (–0.31, 0.46) L | 0 | 0.06 – 0.10 L | 0.85 | 0.77 |

*Egger’s regression and Begg’s rank-correlation p-values are shown; p < 0.05 (none significant except borderline Begg for FVC).
